# A Comparative Study of 0.25% Levobupivacaine, 0.25% Ropivacaine, and 0.25% Bupivacaine in Paediatric Single Shot Caudal Block

**DOI:** 10.1155/2018/1486261

**Published:** 2018-10-31

**Authors:** Jagdeep Sharma, Ruchi Gupta, Anita Kumari, Lakshmi Mahajan, Jasveer Singh

**Affiliations:** ^1^Senior Resident, Dept. of Anaesthesia & Intensive Care, Govt. Medical College & Hospital, Sector-32, Chandigarh, India; ^2^Professor, Dept. of Anaesthesia & Intensive Care, Shri Guru Ram Das Institute of Medical Sciences and Research, Vallah, Amritsar, India; ^3^Assistant Professor, Dept. of Anaesthesia & Intensive Care, Shri Guru Ram Das Institute of Medical Sciences and Research, Vallah, Amritsar, India; ^4^Associate Professor, Dept. of Anaesthesia & Intensive Care, Govt. Medical College & Hospital, Sector-32, Chandigarh, India

## Abstract

**Aim:**

There are limited data comparing levobupivacaine, ropivacaine, and bupivacaine in paediatric patients. So, this study was performed to evaluate the caudal effectiveness of all the three drugs in paediatric patients undergoing infraumbilical surgeries and associated complications with these drugs.

**Material and Methods:**

90 patients of ASA grade I and II posted for elective infraumbilical surgeries were randomly divided into three groups of 30 each. A standardized anaesthetic protocol was used. Patients received 0.25% levobupivacaine in group 1, 0.25% ropivacaine in group 2, and 0.25% bupivacaine in group 3. The effectiveness of block was assessed using caudal effectiveness score. Postoperative pain relief was assessed with modified Hannallah pain score. Haemodynamic parameter monitoring was done. The duration of analgesia and associated complications were studied. Statistical analysis was done using the chi-square test for nonparametric data. Parametric data were analysed using ANOVA for intergroup comparison and Tukey's HSD for intragroup comparison.

**Results:**

Demographic data were comparable. Haemodynamic parameters remained within normal range. Mean caudal effectiveness score in all the three groups was statistically insignificant (*p* > 0.05). The duration of analgesia provided by bupivacaine (145.31 ± 26.17 min) was longer than levobupivacaine (126.15 ± 15.15 min) and ropivacaine (114.68 ± 11.32 min) (*p* < 0.01). Mean postoperative pain scores were lower in group 3 as compared to group 1 and group 2.

**Conclusion:**

We conclude that levobupivacaine and ropivacaine provide similar intraoperative quality with minimal haemodynamic variability and shorter duration of postoperative analgesia without any significant complications when compared with racemic bupivacaine. This trial is registered with CTRI/2018/03/012402.

## 1. Introduction

Anaesthesia for paediatric patients is highly specialised because of the physiological, pharmacological, and psychological differences between children and adults [1]. Regional anaesthesia techniques have become routine interventions in children and infants [2, 3]. Paediatric regional anaesthesia is an excellent technique for balanced intraoperative and postoperative analgesia [4]. Most frequently used technique is epidural block through a caudal approach [5, 6]. It is a useful adjunct during general anaesthesia and for providing postoperative analgesia after genital, lower abdominal, and lower limb operations [7]. It can reduce the inhaled and intravenous anaesthetic requirement, attenuate the stress response to surgery, facilitate a rapid smooth recovery, and provide good immediate postoperative analgesia [8, 9]. Racemic bupivacaine is the most commonly used local anaesthetic. Various single enantiomeric drugs such as levobupivacaine and ropivacaine are now being used, with concerns regarding cardiac toxicity of racemic bupivacaine. Very few studies in the literature had compared all the three drugs together, so this study was planned. It was conducted to compare the caudal effectiveness of the three drugs as the primary outcome and duration of analgesia, associated complications, and haemodynamic variables as secondary outcome.

## 2. Material and Methods

A prospective randomized, double blind study was conducted on 90 patients of ASA grade I and II of either sex aged 1 to 10 years, undergoing infraumbilical surgery as circumcision, herniotomies, orchidopexies, etc. after approval from the hospital ethics committee.

After taking informed written consent, the patients were randomly divided into three groups of 30 each. Randomisation was performed by the computer-generated random number table. For having a power of study more than 80%, the required sample size was calculated to be approximately 90 patients through a pilot study. It was calculated using the number of groups, mean difference of the caudal effectiveness scores and duration of analgesia, standard deviation, and Z power table.

  Group 1: the patients received 1 ml/kg of 0.25% levobupivacaine
  Group 2: the patients received 1 ml/kg of 0.25% ropivacaine
  Group 3: the patients received 1 ml/kg of 0.25% bupivacaine

### 2.1. Exclusion Criteria

Patients with following problems were excluded from the study: refusal by parents or guardian, preexisting neurological disease, hypersensitivity to local anaesthetics under study, bleeding diathesis or coagulation disorder, local sepsis at the site of puncture, and technical problems such as persistent paresthesias or bloody/CSF tap.

The haemodynamic parameters were noted in the preoperative room. All children were premedicated with syrup midazolam 0.5 mg/kg half an hour before surgery. On arrival in the operating room after attaching standard monitoring, all patients were induced with oxygen, nitrous oxide, and sevoflurane, an appropriate sized cannula was inserted, and the intravenous line was started with Isolyte P. The patients were kept on spontaneous ventilation on bag and mask. Lateral decubitus position was made in patients, and caudal block was given after confirming the space with the “Whoosh” test. The study drug was injected. The patients were made to lie in supine position with spontaneous ventilation and anesthesia maintained with oxygen, nitrous oxide, and sevoflurane. Caudal effectiveness score was assessed intraoperatively before beginning of the surgical procedure (Table 1). Surgery was allowed after achieving caudal efficacy.

This would be graded as follows:

  1 = ineffective block
  2 = partial block
  3 = complete block

Continuous intraoperative monitoring will be done, and haemodynamic parameters were recorded every 5 minutes. The patients were shifted to the postanaesthesia care unit. The pain relief was assessed with modified Hannallah pain score [10] (Table 2) in the recovery room and ward every 15 minutes. The duration of absolute analgesia was taken as time from injection of local anaesthetic to modified Hannallah pain score of more than or equal to 4. Injectable paracetamol 10 mg/kg was given as rescue analgesia as required.

Postoperatively vitals in the form of heart rate, respiratory rate, systolic and diastolic blood pressure, and oxygen saturation were monitored. The presence of other adverse events such as bradycardia, respiratory depression, urinary retention, retching, vomiting, and fever was evaluated.

At the end of study, decoding of groups and the data compilation was done. Statistical analysis was done using the chi-square test for nonparametric data. Parametric data were analysed using ANOVA for intergroup comparison and Tukey's HSD for intragroup comparison. *p* value of less than 0.05 was considered significant and less than 0.001 as highly significant. All statistical data analysis was performed using SPSS software (Version 22).

## 3. Results

**Figure 1:**
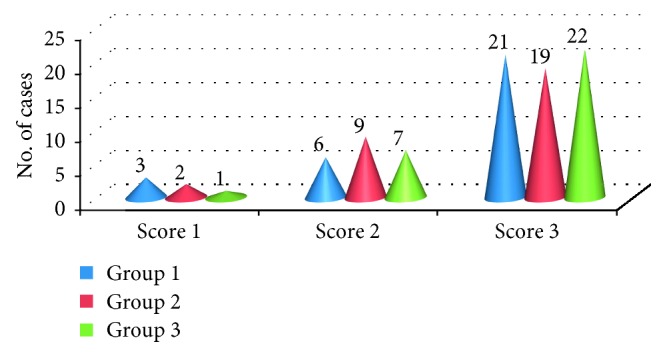
Caudal effectiveness score of the three drugs: group 1 (levobupivacaine); group 2 (ropivacaine); group 3 (bupivacaine).

**Figure 2:**
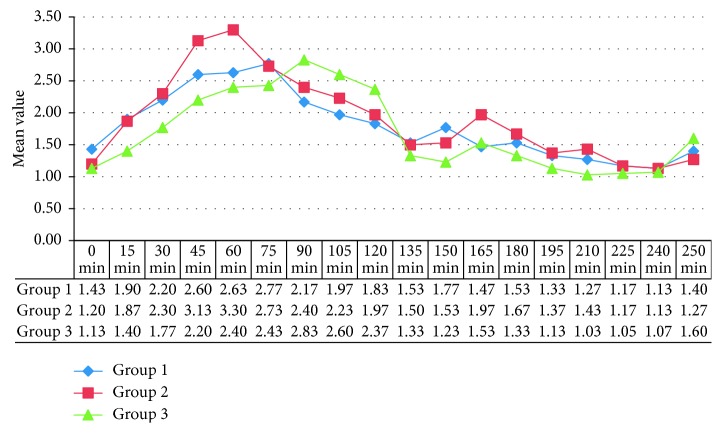
Mean modified Hannallah pain scores of the three drugs: group 1 (levobupivacaine); group 2 (ropivacaine); group 3 (bupivacaine) over the observation period.

There was no statistically significant difference in all the three groups with regard to age, sex, weight, ASA physical status, and duration of surgery as shown in (Table 3). Mean baseline vitals like heart rate, systolic and diastolic blood pressure, respiratory rate, and oxygen saturation were comparable in all the three groups (Table 3). The mean duration of surgery in all the three groups was comparable. Haemodynamic parameters, namely, heart rate, systolic and diastolic blood pressure, respiratory rate, and oxygen saturation, remained within normal range during the observation period, and the difference in all the three groups was statistically insignificant. The difference in mean caudal effectiveness score (CES) in all the three groups was statistically insignificant (*p* > 0.05). 21 patients in group 1 (levobupivacaine), 19 patients in group 2 (ropivacaine), and 22 patients in group 3 (bupivacaine) had a caudal effectiveness score of 3 (Figure 1). Mean postoperative pain scores as assessed with modified Hannallah pain score were lower in the bupivacaine group as compared to the levobupivacaine and ropivacaine groups during 1st hour postoperatively (Figure 2). The duration of postoperative analgesia in group 1 (levobupivacaine) was 126.15 ± 15.15 min (2.10 ± 0.25 hr), group 2 (ropivacaine) was 114.68 ± 11.32 min (1.91 ± 0.18 hr), and group 3 (bupivacaine) was 145.31 ± 15.15 min (2.42 ± 0.43 hr). The difference in duration of postoperative analgesia was statistically highly significant (*p* < 0.001). Levobupivacaine and ropivacaine provided similar duration of postoperative analgesia. Bupivacaine provided longer duration of analgesia than levobupivacaine and ropivacaine.

**Figure 3:**
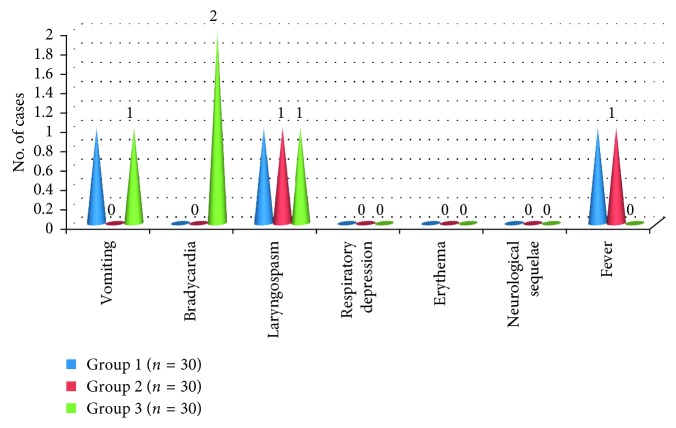
Various complications encountered in all the three groups: group 1 (levobupivacaine); group 2 (ropivacaine); group 3 (bupivacaine).

Three patients, one in each, had laryngospasm intraoperatively. It was managed adequately. Two patients in group 3 (bupivacaine) had bradycardia intraoperatively, which was managed with intravenous atropine. None of the patients in all the three groups had any significant postoperative complications. No neurological sequelae were seen in any patient in all the three groups (Figure 3).

## 4. Discussion

In paediatric patients, although general anaesthesia is the commonly used technique, regional anaesthesia has gained considerable popularity. The primary advantage of regional supplementation is lowering the requirement of general anaesthesia intraoperatively and providing good postoperative pain relief. Caudal anaesthesia is considered as one of the most popular regional blocks performed in children for infraumbilical surgeries. It is safe, reliable, easy to administer, and not associated with any significant complications.

Intraoperative analgesic efficacy of the block was assessed with caudal effectiveness score which include the autonomic component in the form of heart rate and motor component as limb movements [11]. In children undergoing infraumbilical surgery, caudal levobupivacaine 0.25% produced similar intraoperative analgesic efficacy compared to ropivacaine 0.25% and bupivacaine 0.25% [12] similar to this study. Bupivacaine, an amino amide local anaesthetic, is the most commonly used drug in single shot caudal block. It causes more prolonged caudal blockade as compared to single enantiomers like ropivacaine and levobupivacaine. It also causes more prolonged motor blockade as compared to the single enantiomeric drugs [11, 12]. Ropivacaine causes least motor blockade because of its differential action on A delta-fibres than A beta-fibres responsible for motor activity. Bupivacaine tends to be having more cardio depressant effects at similar doses as compared to levobupivacaine and ropivacaine. The three drugs cause minimal variation on haemodynamic parameters. Bupivacaine has an average duration of action of 3.5 to 6 hr given in peridural block. Ivani et al. in their study found the duration of analgesia provided by bupivacaine was 233 min (3.80 hr) [13]. But others have found it to be 2-3 hr as was in this study [12]. Praveen et al. compared 0.25% levobupivacaine and 0.25% ropivacaine for caudal epidural analgesia in paediatric patients. The duration of analgesia provided by both the drugs is statistically similar in both the groups, as in our study, although with longer duration (330 ± 9.54 min in levobupivacaine and 312.67 ± 5.56 min in ropivacaine) as compared with ours [14]. The difference in results among these study results can be due to difference in study age groups involved and induction/maintenance techniques of anaesthesia intraoperatively. Ropivacaine produces 3 to 6 hr of analgesia [15], whereas some authors have reported it to be 1 to 2 hr [11]. Levobupivacaine provide 3 to 6 hr of analgesia [15], whereas others have reported it to be 1 to 2 hr [11] similar to this study. On literature review, the majority of studies have shown the duration of analgesia provided by all the three drugs is quite similar, contrary to our study. The rate of serious complications reported had been 1/40,000 and the total complication rate 1.5/1000. During caudal block, the most frequent complications (due to the technique) encountered were vessel perforation (1.6%–10.6%) and subcutaneous infiltration (5%–19%). Although the local anaesthetics use is quite safe and effective, they may produce systemic toxic reactions affecting the brain and heart. Various complications such as allergic reactions, urinary retention, nausea and vomiting, headache, and dizziness can occur.

We did not encounter any such complications. We had three patients who developed intraoperative laryngospasm which can be due to stimulation in light plane of anaesthesia. Two patients had bradycardia in group 3 which can be attributed to the cardiac effect of systemic absorption of bupivacaine. None of the three drugs were associated with any significant complications. Limitation of our study was that we did not use the awareness monitoring under anesthesia (BIS).

## 5. Conclusion

Thus to conclude, levobupivacaine, bupivacaine, and ropivacaine were effective and safe in terms of stable haemodynamic profile, intraoperative quality, and no significant complications when they were given to patients undergoing infraumbilical surgery under caudal block. Contrary to other studies where these three drugs have shown similar effects, we found that bupivacaine provides longer duration of analgesia than levobupivacaine and ropivacaine, so it needs further studies to substantiate this.

## Figures and Tables

**Table 1:** Caudal effectiveness score.

Score	Definition
1	Able to reduce sevoflurane concentration, heart rate >20% of baseline, along with limb movements
2	Able to reduce sevoflurane concentration, heart rate >20% baseline, with no movements
3	Sevoflurane concentration stopped, minimal or no change in heart rate, no movement on stimulation

**Table 2:** Modified Hannallah pain score (MHPS) [10].

Observation	Criteria	Points
Blood pressure	±10% preop	0
	>20% preop	1
	>30% preop	2

Crying	Not crying	0
	Crying but responds to tender loving care (TLC)	1
	Crying and does not responds to TLC	2

Movement	None	0
	Restless	1
	Thrashing	2

Agitation	Patient asleep or calm	0
	Mild	1
	Hysterical	2

Posture	No special posture	0
	Flexing legs and thighs	1
	Holding scrotum or groin	2

Complains of pain (where appropriate by age)	Asleep or states no pain	0
	Cannot localize	1
	Can localize	2

**Table 3:** Demographic distribution and baseline vitals.

	Group 1	Group 2	Group 3	Statistical analysis
Age (years)	3.78 ± 3.02	4.48 ± 3.24	4.04 ± 3.24	0.631
Sex (F : M)	1 : 29	1 : 29	3 : 27	0.429
Weight (kg)	12.33 ± 5.66	12.96 ± 5.39	11.50 ± 5.39	0.656
Duration of surgery	48.83 ± 19.14	45.83 ± 19.14	48.60 ± 19.92	0.745
Heart rate	116.57 ± 14.31	115.97 ± 10.96	115.87 ± 15.89	0.45
Systolic blood pressure	104.37 ± 3.13	104.27 ± 3.47	103.87 ± 3.44	0.51
Diastolic blood pressure	59.43 ± 3.97	57.47 ± 5.89	59.99 ± 5.37	0.34

Data are mean ± SD. *p* > 0.05 insignificant; *p*<0.05 significant.

## Data Availability

Data is available on request. Requests for access to these data should be made to Dr. Jagdeep Sharma, MBBS, MD Senior Resident, Anesthesia and Critical Care, Government Medical College and Hospital, Sector 32, Chandigarh, India, e-mail: Snehlatas222@gmail.com.
